# Effect of the interaction between MiR‐200b‐3p and *DNMT3A* on cartilage cells of osteoarthritis patients

**DOI:** 10.1111/jcmm.13152

**Published:** 2017-03-27

**Authors:** Jian Wu, Yunjuan Tao, Anquan Shang, Weiwei Wang, Yujie Zhang, Liqing Hu, Jun Wang, Yuan Wang, Naizhou Guo

**Affiliations:** ^1^ Department of Laboratory Medicine The First People's Hospital of Yancheng City Yancheng Jiangsu China; ^2^ Yancheng TCM Hospital Affiliated To Nanjing University of Chinese Medicine Yancheng Jiangsu China; ^3^ Clinical Medicine School Ningxia Medical University Yinchuan Ningxia China; ^4^ Department of Laboratory Medicine The Sixth People's Hospital of Yancheng City Yancheng Jiangsu China; ^5^ Department of Laboratory Medicine The First Hospital of Ningbo City Ningbo Zhejiang China; ^6^ Department of Laboratory Medicine The First Affiliated Hospital of Zhejiang Chinese Medicine University Hangzhou Zhejiang China

**Keywords:** osteoarthritis, cartilage cell, miR‐200b‐3p, *DNMT3A*

## Abstract

The aim of this research is to explore the effect of miR‐200b‐3p targeting *DNMT3A* on the proliferation and apoptosis of osteoarthritis (OA) cartilage cells. Quantitative RT‐PCR was performed to analyse the expression of miR‐200b‐3p, *DNMT3A*,*MMP1*,*MMP3*,*MMP9*,*MMP13* and *COL II* in normal and OA cartilage tissues. The dual‐luciferase reporter assay and Western blot assay were conducted to confirm the targeting relationship between miR‐200b‐3p and *DNMT3A*. We also constructed eukaryotic expression vector to overexpress miR‐200b‐3p and *DNMT3A*. We detected the expression level of *MMPs* and *COL II* in stable transfected cartilage cells using RT‐PCR and Western blot. Cell proliferation and apoptosis were evaluated using the MTS, pellet culture and Hoechst 33342 staining method. Finally, we explored the effect of miR‐200b‐3p targeting *DNMT3A* on the proliferation and apoptosis of OA cartilage cells. The results of RT‐PCR indicated that both miR‐200b‐3p and *COL II* were down‐regulated in OA cartilage tissues, while the expression of *DNMT3A* and *MMPs* was up‐regulated in OA cartilage tissues. The expressions of *DNMT3A*,*MMPs* and *COL II* detected by Western blot showed the same trend of the results of RT‐PCR. The dual‐luciferase reporter assay and Western blot assay confirmed the targeting relationship between miR‐200b‐3p and *DNMT3A*. In overexpressed miR‐200b‐3p cartilage cells, *DNMT3A* and *MMP*s were significantly down‐regulated, *COL II* was significantly up‐regulated, cell viability was enhanced and apoptosis rate was decreased (*P* < 0.05). In overexpressed *DNM3T* cartilage cells, *MMPs* were significantly up‐regulated, *COL II* was significantly down‐regulated, cell viability was weakened and apoptosis rate was increased (*P* < 0.05). MiR‐200b‐3p inhibited the secretion of *MMPs*, promoted the synthesis of *COL II* and enhanced the growth and proliferation of OA cartilage cells through inhibiting the expression of *DNMT3A*.

## Introduction

Osteoarthritis (OA) is a cartilage disease which may cause local inflammatory responses, tissue damages and cartilage structure abnormality 1, 2. OA is classified as a heterogeneous disease with multifactorial aetiology, including biochemical abnormalities and immunologic reactions 2. OA affects more than 10% of the population, particularly the aged 2, and it is usually controlled by non‐steroidal anti‐inflammatory drugs (NSAIDs) or cyclooxygenase (COX)‐2 inhibitors which are able to provide patients with symptom relief. However, side effects associated with the above medications have been observed in OA patients 3. As a result of this, researchers started to seek for new therapeutic strategies in order to overcome these issues.

Although the pathogenesis of OA is still unclear, the damage of extracellular matrix has been verified to be one of an important event resulting in the progression of OA. For instance, previous studies have indicated that degraded enzymes, matrix metalloproteinases (*MMPs*), accumulation of inflamed synovium and the articular chondrocytes may trigger the degradation of the extracellular matrix of cartilage 4, 5. Apart from that, collagens especially type II collagen (*COL II*) which accounts for a large proportion of major structural components in the matrix of the chondrocytes is another key element in damaged extracellular matrix 6. Researchers also suggested that the apoptosis of chondrocytes may play an essential role in the pathogenesis of OA 7.

Increasing evidence has suggested that the aetiology of OA may be associated with the alterations of gene expressions in chondrocytes 8, 9, 10. MicroRNAs (miRNAs) are endogenous small non‐coding RNAs and it is believed that they regulate about one third of the genes *via* binding to the miRNAs 11, 12. Hundreds of miRNAs have been revealed to be associated with many human diseases including OA 8, 9, 10. For instance, Miyaki *et al*. have reported that microRNA‐140 is dysregulated in OA and the inhibition of miR‐140 causes the progression of OA‐like changes 13. In addition, a study carried out by Akhtar *et al*. suggested that miR‐27b is a post‐transcriptional regulator of matrix metalloproteinase‐13 (*MMP‐13*) 14. Although there is a large number of miRNAs which have been linked with OA, evidence with respect to the association between miR‐200b‐3p and OA has not been revealed in the current literature. A series of studies have recognized miR‐200b‐3p as a regulatory factor for cell proliferation and mobility in many cancers 15, 16, 17, but its potential function in OA development remains unknown.

DNA methyltransferase 3 alpha (*DNMT3A*) is a member of the DNA methyltransferase enzymes family (*DNMTs*) and it participates in the catalysis of DNA methylation which is responsible for embryonic development, gene expression regulation as well as many other vital biological activities and processes 18, 19. *DNMT3A* is generally believed to be amplified in early embryonic cells and to be responsible for de novo CpG methylation 20. Moreover, *DNMT3A* has been found to be associated with some human neoplasms 21, 22. On the other hand, it has been reported that *DNMT3A* is potentially regulated by certain miRNAs including miR‐200b 23.

Since miRNAs are potential regulators of gene expressions and they tend to impact on the progression of OA, we performed a series of experiments to study the potential functions of miR‐200b‐3p in the survival of OA chondrocytes. Besides, we also measured the expression level of *MMPs* and *COL II* in OA chondrocytes and evaluated the effects of miR‐200b‐3p on matrix synthesis. Furthermore, we conducted experiments to confirm whether miR‐200b‐3p is able to target *DNMT3A*. We designed the above experiments in order to enhance the understanding of the corresponding aetiology of OA so that alternatives to NSAIDs or cyclooxygenase (COX)‐2 inhibitors can be developed accordingly.

## Materials and methods

### Patients and tissue specimens

OA cartilage specimens were obtained from a total of 27 patients who accepted joint inflammatory arthroscopy cleaning operation or the knee joint clear operation (the ICRS macroscopic score of these patients was level I with superficial lesions, fissures and cracks, soft indentation or level II with fraying, lesions extending down to <50% of cartilage depth) from 2013 to 2015 in the First Affiliated Hospital of Zhejiang Chinese Medicine University and the First People's Hospital of Yancheng City. The mean age of these 27 patients was 57.6 years. Normal cartilage specimens were obtained from a total of 14 emergency traumatic amputated patients with an average age of 34.6 years old. All patients with rheumatoid arthritis and septic arthritis were excluded in our study. Full‐thickness cartilage and a few subchondral bony in the femoral condyle of all patients were cut out before tissue examination. Then, the samples were put into liquid nitrogen at once and stored for cryopreservation. Written informed consents were, respectively, obtained from each patient and approval of the study was also obtained from the ethic committee of the First Affiliated Hospital of Zhejiang Chinese Medicine University and the First People's Hospital of Yancheng City.

### Cell culture

After the fibrous connective tissues were removed under aseptic conditions, we cut the cartilage into the size of 1 mm^3^. PBS containing sodium penicillin and gentamicin double fluid resistance was used to wash the resulting tissues for several times. Subsequently, 0.25% trypsin was added using the ratio of 1:5 and the corresponding product was digested for 30 min. Then, 0.2% type II collagenase was added according to the ratio of 1:5. After 16‐hrs digestion, cells were collected every four hours. Cell suspension was centrifuged and filtered using a 200‐mesh sieve filter at a pace of 1000 r/min for 5 min. The supernatant was discarded and a final wash was performed for three times using the complete medium containing 10% foetal bovine serum (FBS). Cells were seeded at a density of 1 × 10^5^/ml and incubated under 37°C, 5% CO_2_ condition.

### Cell transfection

We separately inserted miR‐200b‐3p mimics (Applied Biosystems, Foster City, CA, USA) and *DNMT3A* (Applied Biosystems) into miRNA Select pEGP‐miR (Cell Biolabs, SanDiego, CA, USA) and PCMV6‐XL5 (OriGene, Rockville, MD, USA) for constructing the overexpressed plasmids of pEGP‐miR‐200b‐3p and PCMV6‐*DNMT3A*. *DNMT3A* siRNA was purchased from GenePharma, Shanghai, China. The following experimental groups were constructed: negative control group (NC group: cells simultaneously transfected with pEGP and PCMV6 empty vector), miR mimics group (cells simultaneously transfected with pEGP‐miR‐200b‐3p and PCMV6 empty vector plasmid), *DNMT3A* group (cells simultaneously transfected with pEGP empty vector and PCMV6‐*DNMT3A*), si‐*DNMT3A* group (cells simultaneously transfected with pEGP empty vector and *DNMT3A* siRNA) and miR mimics + *DNMT3A* group (cells simultaneously transfected with pEGP‐miR‐200b‐3p and PCMV6‐*DNMT3A*). The above transfection was conducted using the Lipofectamine 2000 reagents (Invitrogen, Carlsbad, CA, USA).

### Real‐time Quantitative PCR (RT‐PCR)

Total RNA was isolated with TRIzol reagent (Thermo Fisher Scientific, Waltham, MA, USA) and was reverse‐transcribed into cDNA before the implementation of qRT‐PCR analysis. RT‐PCR for miRNAs was performed with a miR‐200b‐3p TaqMan MicroRNA assay. U6 miRNA was used as a housekeeping control. The expression levels of *DNMT3A*,* MMP1*,* MMP3*,* MMP9*,* MMP13* and *COL II* were analysed using RT‐PCR and normalized to the GAPDH. Fold changes in gene expression were quantified by using the 2^−ΔΔCt^ method. The corresponding primer sequences are listed in Table 1.

**Table 1:** RT‐PCR primer sequences

	Primer sequence
MiR‐200b‐3p	F: 5′‐GCTGCTGAATTCCATCTAATTTCCAAAAG‐3′
MiR‐200b‐3p	R: 5′‐TATTATGGATCCGCCCCCAGGGCAATGGG‐3′
U6	F: 5′‐CTTCGGCAGCACATATAC‐3′
U6	R: 5′‐GAACGCTTCACGAATTTGC‐3′
DNMT3A	F: 5′‐CATCTGCATCTCCTGTGGG‐3′
DNMT3A	R: 5′‐GCAGTTTTGGCACATTCCTC‐3′
MMP1	F: 5′‐AAAATTACACGCCAGATTTGCC‐3′
MMP1	R: 5′‐GGTGTGACATTACTCCAGAGTTG‐3′
MMP3	F: 5′‐CTGGACTCCGACACTCTGGA‐3′
MMP3	R: 5′‐CAGGAAAGGTTCTGAAGTGACC‐3′
MMP9	F: 5′‐AGACCTGGGCAGATTCCAAAC‐3′
MMP9	R: 5′‐CGGCAAGTCTTCCGAGTAGT‐3′
MMP13	F: 5′‐TTGCAGAGCGCTACCTGAGATCAT‐3′
MMP13	R: 5′‐TTTGCCAGTCACCTCTA AGCCGAA‐3′
COL II	F: 5′‐GGTGGAGCAGCAAGAGCAA‐3′
COL II	R: 5′‐AGTGGACAGTAGACGGAGGAAA‐3′
GAPDH	F: 5′‐TGGTCACCAGGGCTGCTT‐3′
GAPDH	R: 5′‐AGCTTCCCGTTCTCAGCC‐3′

### Dual‐luciferase reporter assay

As suggested by the TargetScan system (http://www.targetscan.Org/), *DNMT3A* is one potential target of miR‐200b‐3p. The corresponding sequences contained the miR‐200b‐3p wild‐type target region of *DNMT3A* 3′UTR and its mutant (Sangon, Shanghai, China), which were inserted into the PCMV6 vector (Promega, Madison, WI, USA) for constructing the corresponding PCMV6‐wt plasmid and PCMV6‐Mut plasmid. The cells were divided into four groups according to different transfections: cells simultaneously transfected with pEGP‐miR‐200 and PCMV6‐wt were included in the miR‐wt group, cells simultaneously transfected with pEGP‐miR‐200 and PCMV6‐mut were included in the miR‐mut group, cells simultaneously transfected with pEGP empty vector and PCMV6‐wt were included in the NC‐wt group and cells simultaneously transfected with pEGP empty vector and PCMV6‐mut were included in the NC‐mut group. The corresponding medium in each group was replaced after six hours and the relative luciferase activity was determined 24 hrs after the transfection.

### Pellet culture

The formation of cartilage‐like tissue in vitro was investigated in high‐density pellet cultures. Briefly, 5 × 10^5^ cells in 0.5 ml of DMEM containing gentamicin (50 mg/ml), L‐glutamine (300 mg/ml), amphotericin B (2.5 mg/ml), ascorbic acid (50 μg/ml) and 0.1% FBS were centrifuged at 1200 r/min for 5 min. in 15‐ml polypropylene tubes. The cell pellets were cultured for 3 weeks, and the medium was changed twice a week. Cells were stained for 10 min. in toluidine blue and macroscopic images were captured using a stereomicroscope (MVX‐10 MacroView Systems, Olympus, Tokyo, Japan) equipped with a DP71 camera (Olympus).

### Western blots

After 48 hrs of transfection, cells were lysed on ice‐cold RIPA buffer (Solaibo, China) for total protein extraction. Cell lysate was separated by SDS‐PAGE, and the protein concentrations were quantified by using the BCA Kit (Sigma‐Aldrich, St Louis, MO, USA). Protein extracts were transferred to PVDF membranes, blocked with 5% non‐fat milk in TBST and subsequently incubated with DNMT3A, MMP1, MMP3, MMP9, MMP13, COL II, ERK1/2, p‐ERK1/2 and GAPDH antibodies at 4°C overnight. Then, the resulting membranes were mixed with secondary antibodies and incubated for 1 hr at room temperature. After that, enhanced chemiluminescence (ECL) was conducted using the detection reagents (GE Healthcare, Chalfont St Giles, Bucks, UK) for further analysis.

### MTS

Cells of different groups were seeded in a 24‐well plate at the density of 1 × 10^3^ per well. The absorptions of cells were measured at 492 nm, and the corresponding measurements were performed at 24, 48, 73 and 96 hrs by using a Cell Titer 96^®^ AQueous One Solution Cell Proliferation Assay (MTS) (Promega, Beijing, China).

### Hoechst 33342 staining

Cells transfected for 24 hrs were collected and the old medium was discarded after the transfection. After that, 2 ml 1640 medium containing 2% FBS was added to the cells. Then, Hoechst 33342 was added until the density reached the level of 5 μg/ml and the resulting product was incubated for 90 min. at 37°C in the dark. PBS was used to wash the resulting product twice and another 1 ml 1640 medium containing 2% FBS was added. Finally, we randomly selected 5 × 100 field for the purpose of cell apoptosis observation which was conducted under the fluorescence microscope.

### Statistical analysis

Data obtained from the above experiments were presented as mean ± S.D. The *t*‐test or analysis of variance (anova) was conducted for comparing data in different groups provided that the normality assumption was not violated. Alternatively, the nonparametric rank‐sum test was carried out in the case that the normality assumption is violated. A *P*‐value of less than 0.05 provided evidence for statistical significance.

## Results

### MiR‐200b‐3p and DNMT3A in normal and OA cartilage tissues/cells

**Figure 1:**
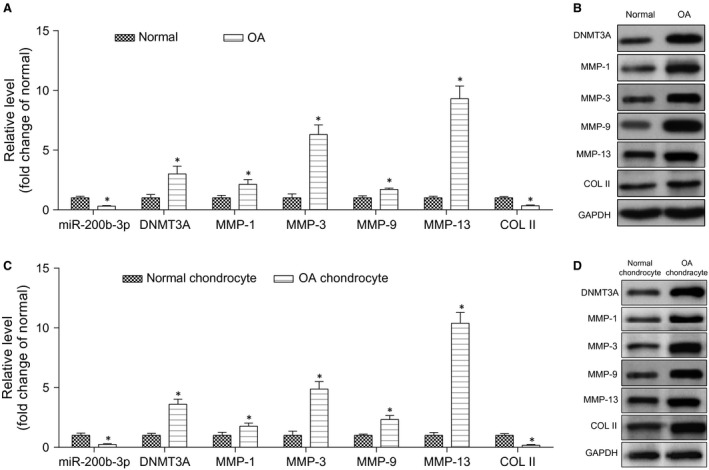
The expression levels of miR‐200b‐3p, *DNMT3A*,*MMPs* and *COL II*. (**A**) RT‐qPCR detected the expression levels of miR‐200b‐3p, *DNMT3A*,*MMPs* and *COL II* in normal and OA cartilage tissues. *P < 0.05 compared to the normal group. (**B**) Western blot was used to detect the expression of *DNMT3A*,*MMPs* and *COL II* in normal and OA cartilage tissues. (**C**) RT‐qPCR detected these expression levels in normal and OA cartilage cells. *P < 0.05 compared to the normal chondrocyte group. (d) Western blot was used to detect the expression of *DNMT3A*,*MMPs* and *COL II* in normal and OA cartilage cells.

As suggested by Figure 1A and B, OA cartilage tissues exhibited a significant decrease in the expression of miR‐200b‐3p compared to that in normal human cartilage tissues, which was further confirmed by in situ hybridization (Figure 1). However, the expression of *DNMT3A* in OA cartilage tissues was significantly elevated compared to that in normal cartilage tissues (*P* < 0.05). Besides that, OA cartilage tissues exhibited significantly higher mRNA expression of *MMP1*,* MMP3*,* MMP9* and *MMP13* compared to those in normal cartilage tissues whereas the mRNA expression of *COL II* was significantly down‐regulated in OA cartilage tissues (*P* < 0.05). The above trend was confirmed by Figure 1C which compared these expressions between normal and OA chondrocytes. OA cartilage cells exhibited significantly lower levels of miR‐200b‐3p and *COL II* compared to those in normal chondrocytes. On the other hand, the expression levels of *DNMT3A*,* MMP1*,* MMP3*,* MMP9* and *MMP13* were significantly up‐regulated in OA cartilage cells as compared to normal chondrocytes (*P* < 0.05). The protein expression levels of *DNMT3A*,* MMP1*,* MMP3*,* MMP9* and *MMP13* detected by Western blot showed the same trend of the mRNA expression levels in Figure 1D.

### MiR‐200b‐3p targeted and suppressed DNMT3A expression

**Figure 2:**
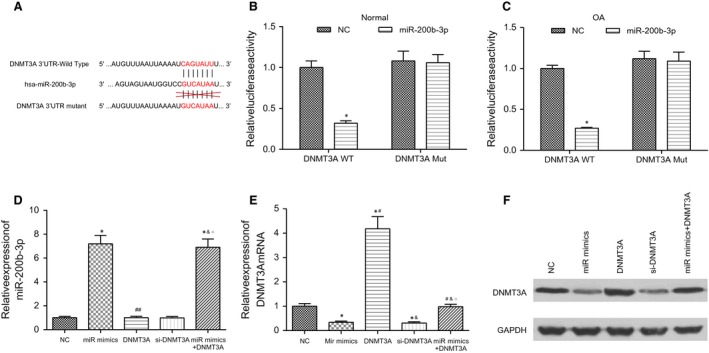
Transfection of MiR‐200b‐3p into cartilage cells inhibited the expression of *DNMT3A*. (**A**) Constructed *DNMT3A* 3′UTR Wide Type (wt) and *DNMT3A* 3′UTR mutant (mut) in order to ensure that miR‐200b‐3p contained binding sites only with *DNMT3A* 3′UTR wt; (**B**), (**C**) miR‐200b‐3p combining with *DNMT3A* 3′UTR wt significantly reduced the luciferase activity of normal and OA cartilage cells. *P < 0.05 compared to the NC group. (**D**), (**E**) RT‐PCR analysed the expression levels of miR‐200b‐3p and DNMT3A mRNA in all transfected groups. *P < 0.05 compared to the NC group. ^#^ *P* < 0.05 compared to the miR mimics group. ^&^ *P* < 0.05 compared to the *DNMT3A* group. ^○^ *P* < 0.05 compared to the si‐*DNMT3A* group. (**F**) Western blot assay further confirmed that miR‐200b‐3p could suppress the expression of *DNMT3A* in OA cartilage cells. NC: negative control group, cells simultaneously transfected with pEGP and PCMV6 empty vector. MiR mimics: miR‐200b‐3p mimics group, cells simultaneously transfected with pEGP‐miR‐200b‐3p and PCMV6 empty vector plasmid. *DNMT3A*:*DNMT3A* group, cells simultaneously transfected with pEGP empty vector and PCMV6‐*DNMT3A*. Si‐*DNMT3A*:*DNMT3A* siRNA group, cells transfected with *DNMT3A* siRNA. MiR mimics + *DNMT3A*: miR mimics + *DNMT3A* group, cells simultaneously transfected with pEGP‐miR‐200b‐3p and PCMV6‐*DNMT3A*.

As suggested by the TargetScan database (Fig. 2A), the binding sites of miR‐200b‐3p are located in the position of 1441‐1447 contained in the region of *DNMT3A* 3′UTR. The luciferase activity in the pEGP‐miR‐200 + PCMV6‐WT group was significantly decreased compared to that in the NC group (*P* < 0.05). There is no significant difference in the luciferase activity between the pEGP‐miR‐200 + PCMV6‐Mut and NC group (*P* > 0.05, Fig. 2B and C). The above results confirmed that miR‐200b‐3p had direct targeting effects on *DNMT3A*. Results of RT‐PCR suggested that the expressions of miR‐200b‐3p in the miR mimics group and the miR mimics + *DNMT3A* group were significantly elevated compared to that in the NC group (*P* < 0.05, Fig. 2D). The expressions of *DNMT3A* in the miR mimics group and si‐*DNMT3A* group were significantly decreased compared to that in the NC group (*P* < 0.05, Fig. 2E). On the other hand, the *DNMT3A* group exhibited significantly higher expression of *DNMT3A* compared to the NC group (*P* < 0.05, Fig. 2E). The above results verified that miR‐200b‐3p had potential targeting and suppressive effects on the expression of *DNMT3A*. Results of Western blot are displayed in Fig. 2F which revealed that the protein expression levels of *DNMT3A* in the miR‐200b‐3p mimics group and si‐*DNMT3A* group were inhibited compared to that in the NC group, while the transfection of *DNMT3A* triggered a significant up‐regulation of the protein expression of DNMT3A. Therefore, we concluded that miR‐200b‐3p may exhibit targeting and suppressive effects on *DNMT3A*.

### MiR‐200b‐3p inhibited the degradation of cartilage extracellular matrix

**Figure 3:**
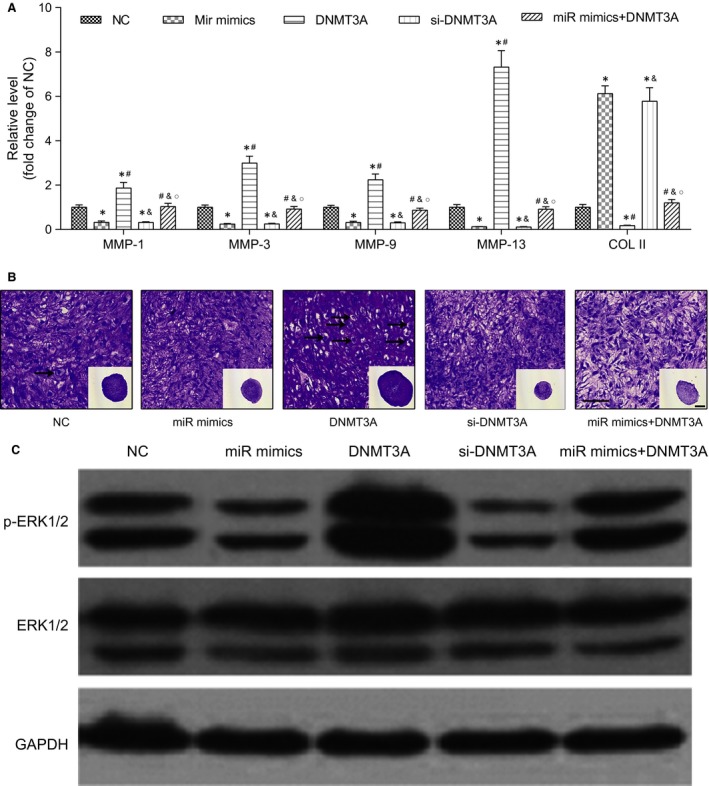
MiR‐200b‐3p inhibited the degradation of cartilage extracellular matrix. (**A**) RT‐PCR analysed the expression levels of *MMPs* and *COL II* in all transfected groups. *P < 0.05 compared to the NC group. ^#^ *P* < 0.05 compared to the miR mimics group. ^&^ *P* < 0.05 compared to the *DNMT3A* group. ^○^ *P* < 0.05 compared to the si‐*DNMT3A* group. (**B**) Pellet culture of chondrocyte (50 μm). (**C**) Western blot was used to confirm the expression of p‐ERK1/2 of the MAPK/ERK pathway. NC: negative control group, cells simultaneously transfected with pEGP and PCMV6 empty vector. MiR mimics: miR‐200b‐3p mimics group, cells simultaneously transfected with pEGP‐miR‐200b‐3p and PCMV6 empty vector plasmid. *DNMT3A*:*DNMT3A* group, cells simultaneously transfected with pEGP empty vector and PCMV6‐*DNMT3A*. Si‐*DNMT3A*:*DNMT3A* siRNA group, cells transfected with *DNMT3A* siRNA. MiR mimics + *DNMT3A*: miR mimics + *DNMT3A* group, cells simultaneously transfected with pEGP‐miR‐200b‐3p and PCMV6‐*DNMT3A*.

As suggested by the results of RT‐PCR (Fig. 3A), the miR mimics group and si‐*DNMT3A* group exhibited significantly lower expression levels of *MMP1*,* MMP3*,* MMP9* and *MMP13* compared to those in the NC group. However, the miR mimics group and si‐*DNMT3A* group exhibited significantly higher expression of *COL II* in reference to the NC group, suggesting the potential inhibition of the degradation of the cartilage extracellular matrix (*P* < 0.05, Fig. 3A). By contrast, the *DNMT3A* group exhibited significantly higher expression of *MMPs* but significantly lower expression of *COL II* in reference to the NC group, indicating evidence for the deterioration of OA.

In pellet cultures of control group, there was a remarkable increase in pellet size in the *DNMT3A* group compared with NC group, whereas there was a significant decrease in the miR mimics group and si‐*DNMT3A* group compared with NC group in day 21. The number of hypertrophy chondrocytes in the *DNMT3A* group also increased, while the number of hypertrophy chondrocytes in the miR mimics and si‐*DNMT3A* groups decreased (Fig. 3B).

### MiR‐200b‐3p affected cartilage extracellular matrix *via* MAPK/ERK pathway

Western blot was used to confirm the expression of ERK1/2 and p‐ERK1/2 of the MAPK/ERK pathway. As shown in Figure 3C, the protein expression levels of p‐ERK1/2 in the miR mimics group and si‐*DNMT3A* group were inhibited compared to that in the NC group, while the *DNMT3A* group triggered a significant up‐regulation of the protein expression of DNMT3A. There was no significant difference of the expression of ERK1/2 among the NC, the miR mimics, the *DNMT3A,* the si‐*DNMT3A* and the miR mimics + *DNMT3A* groups. Thus, we supposed that miR‐200b‐3b/*DNMT3A* affected cartilage extracellular matrix through the MAPK/ERK pathway.

### MiR‐200b‐3p enhanced the viability of cartilage cells and suppressed cell apoptosis

**Figure 4:**
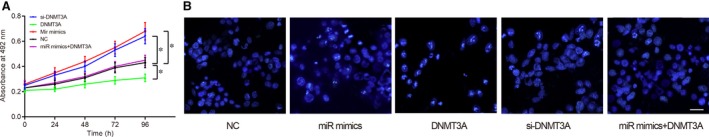
MiR‐200b‐3p enhanced the viability of cartilage cells and suppressed cell apoptosis. (**A**) MTS detected the cell viability in each transfected group: the miR mimics group exhibited significantly improved cell proliferation in reference to the NC group. The *DNMT3A* group exhibited worse cell proliferation than that in the NC group. *P < 0.05 compared to the NC group. (**B**) Hoechst 33342 staining results photograph (inverted phase contrast microscope ×200): the miR mimics group exhibited an improved cell growth and a smaller proportion of apoptotic cartilage cells than that in the NC group; the *DNMT3A* group exhibited a worse cell growth and a higher proportion of apoptotic cartilage cells in reference to the NC group. There was no significant difference in the cell growth pattern or the proportion of apoptotic cartilage cells between the NC and miR mimics + *DNMT3A* group. NC: negative control group, cells simultaneously transfected with pEGP and PCMV6 empty vector. MiR mimics: miR‐200b‐3p mimics group, cells simultaneously transfected with pEGP‐miR‐200b‐3p and PCMV6 empty vector plasmid. *DNMT3A*:*DNMT3A* group, cells simultaneously transfected with pEGP empty vector and PCMV6‐*DNMT3A*. Si‐*DNMT3A*:*DNMT3A* siRNA group, cells transfected with *DNMT3A* siRNA. MiR mimics + *DNMT3A*: miR mimics + *DNMT3A* group, cells simultaneously transfected with pEGP‐miR‐200b‐3p and PCMV6‐*DNMT3A*.

Results of MTS suggested that the miR mimics group and si‐*DNMT3A* group exhibited improved cartilage cell proliferation in reference to the NC group (Fig. 4A). On the other hand, the *DNMT3A* group exhibited significantly less cell proliferation and viability as compared to the NC group (*P* < 0.05). The above results implied that mir‐200b‐3p stimulated the proliferation of cartilage cells by potentially inhibiting the expression of *DNMT3A* expression. Results of Hoechst 33342 staining are displayed in Figure 4B. Living cells exhibited diffuse and homogeneous fluorescence. There appeared to be strong fluorescent dye dense particles block in the apoptotic cell nucleus or cytoplasm. The miR mimics group and si‐*DNMT3A* group exhibited a smaller proportion of apoptotic cartilage cells in reference to the NC group. However, the *DNMT3A* group exhibited a more aggressive cell apoptosis in reference to the NC group. Based on the above results, we suspected that miR‐200b‐3p suppressed cartilage cell apoptosis may through inhibiting the expression of *DNMT3A*.

## Discussion

OA is a degenerative joint disease which causes articular cartilage damage 1. The most apparent symptoms of OA include joint pain and inflammation 12. Statistics has reported that more than 10% patients, especially the elderly, are suffering from this disease 2. At present, therapies for OA patients mainly are aimed to alleviate pain and inflammation. However, no solutions have been discovered to cure this disease 1, 3 and OA medications often cause severe side effects 1, 3. Thus, exploring the pathogenesis of OA has become an essential and urgent task.

Accumulating evidence has indicated that microRNAs are associated with OA progression 8, 9, 10. In our study, miR‐200b‐3p was significantly down‐regulated in OA cartilage tissues and cells. To determine the role of miR‐200b‐3p in OA development, we subsequently performed experiments to evaluate its effects on the survival and matrix synthesis of OA chondrocytes through introducing exogenous miR‐200b‐3p mimics. Furthermore, we explored the possible molecular mechanism induced by miR‐200b‐3p *via* identifying its targets which are associated with OA.

The effects of miR‐200b on certain cancers have been investigated in previous studies. For example, Tang *et al*. have found that the expression of miR‐200b is repressed in the gastric cancer specimens and cell lines 23. Yu *et al*. have reported that miR‐200b restrains the tumour formation of breast cancer 24. In addition to the suppressive effect of miR‐200b on tumour growth, miR‐200b has been found to inhibit the epithelial–mesenchymal transition (EMT) process of glioma cells 16. However, the association between miR‐200b and OA has not been verified in the current literature.

We compared the expression level of miR‐200b‐3p between OA tissues and normal cartilage tissues. Mir‐200b‐3p was significantly down‐regulated in OA tissue samples compared to that in normal ones, and similar results were observed in OA chondrocytes. We also attempted to confirm the role of miR‐200b‐3p in regulating the biological processes of OA chondrocytes. Results of MTS assay suggested that OA chondrocytes in which miR‐200b‐3p mimics was transfected exhibited a higher multiplication rate compared to normal chondrocytes. Besides, the apoptosis of OA chondrocytes with miR‐200b‐3p overexpression showed significant decrease. Therefore, we concluded that overexpression of miR‐200b‐3p could improve the viability of OA chondrocytes.

It is generally believed that miRNAs participate in biological processes through regulating related genes 11. According to the report of Tang *et al*., miR‐200b regulated the progression of gastric cancers by targeting DNA methyltransferases including *DNMT3A* gene. Thus, we proposed that *DNMT3A* might also involve in the miR‐200b‐3p regulation mechanism in OA progression. *DNMT3A* has been previously reported to exhibit an increased expression level in early embryonic cells, and to catalyse the methylation of the CpG sites which is closely related to anti‐oncogene silencing 20, 22. Nevertheless, few studies available devoted to understanding the potential functions of *DNMT3A* in OA pathogenesis except for Sesselmann *et al*. who demonstrated that there was no significant difference in *DNMT3A* expression levels between OA chondrocytes and normal ones 25. On the contrary, our study showed significant amplification of *DNMT3A* mRNA expression in OA tissues as well as in OA chondrocytes in comparison with normal ones. Then, we managed to analyse the biological functions of *DNMT3A* in OA chondrocytes by overexpressing it in cells. The opposite effects were observed in OA chondrocytes with *DNMT3A* overexpressed compared with those transfected with miR‐200b‐3p mimics in our study. Higher level of *DNMT3A* restricted the proliferation and anti‐apoptosis of OA chondrocytes. To further determine the specific regulatory relationship between miR‐200b‐3p and *DNMT3A*, we employed the dual‐luciferase assay and found that miR‐200b‐3p did effectively bind the 3′UTR region of *DNMT3A* mRNA. RT‐qPCR and Western blot analyses also showed that the *DNMT3A* expression level was suppressed in OA chondrocytes with overexpressed miR‐200b‐3p. On the other hand, up‐regulation of *DNMT3A* could counteract the effect of overexpressed miR‐200b‐3p on the viability and apoptosis of OA chondrocytes. All in all, we were able to demonstrate that *DNMT3A* is one of the miR‐200b‐3p targets and up‐regulation of miR‐200b‐3p could improve the viability of OA chondrocytes *via* directly suppressing the *DNMT3A* expression.

Since the cartilage degradation is one of the central characteristics of OA 26, we subsequently investigated the expression of genes which are related to matrix degradation. Existing researches have manifested that matrix metalloproteinases (*MMPs*) accumulate in inflamed tissues and specifically cleave the triple helix of type II collagen (*COL II*) 4, 26. In our study, up‐regulation of miR‐200b‐3p significantly suppressed the levels of *MMPs* while increased the expression of *COL II* expression level, indicating that miR‐200b‐3p could serve as a repair factor for OA cartilage. Moreover, overexpressing *DNMT3A* offset the recovery effect of miR‐200b‐3p on OA cartilage. The above findings provide new insights for managing OA through targeting the potential candidate of miR‐200b‐3p. However, further evidence should be obtained in order to verify the above conclusions since we did not evaluate the correlation between the expressions of miR‐200b‐3p and *DNMT3A* using a large sample size.

In summary, our study demonstrated that miR‐200b‐3b is a potential candidate which is able to improve the viability of chondrocytes and impede the matrix degradation *via* its direct inhibitory role in *DNMT3A*.

## Conflicts of interest

None.

## Author contribution

JW, YT and AS performed the research; WW and YZ analysed and interpreted the data; JW, YT, LH and JW conducted the experiments; JW, YT, YW and ZG designed the research study and wrote the paper; YW and NG sponsored this study.

## Supporting information


**Figure S1** The miR‐200b‐3p expression levels were determined in normal cartilage and OA cartilage by *in situ* hybridization (inverted phase contrast microscope ×200). (**A**) The expression of miR‐200b‐3p in normal cartilage tissues was detected by *in situ* hybridization. (**B**) The expression of miR‐200b‐3p in OA cartilage tissues was detected by *in situ* hybridization.
